# Encapsulation Capacity of β-Cyclodextrin Stabilized Silver Nanoparticles towards Creatinine Enhances the Colorimetric Sensing of Hydrogen Peroxide in Urine

**DOI:** 10.3390/nano11081897

**Published:** 2021-07-24

**Authors:** Abdelaziz Elgamouz, Chahlaa Nassab, Alaa Bihi, Somaya A. I. Mohamad, Aisha H. S. A. Almusafri, Salman S. Alharthi, Sarah A. E. Abdulla, Shashikant P. Patole

**Affiliations:** 1 Department of Chemistry, College of Sciences, University of Sharjah, Sharjah P.O. Box 27272, United Arab Emirates; U19103696@sharjah.ac.ae (C.N.); abihi@sharjah.ac.ae (A.B.); u00041765@sharjah.ac.ae (S.A.I.M.); u00042707@sharjah.ac.ae (A.H.S.A.A.); 2 Department of Chemistry, College of Science, Taif University, P.O. Box 11099, Taif 21944, Saudi Arabia; s.a.alharthi@tu.edu.sa; 3 Mohamed Bin Zayed University for Humanities, Al Muroor Street, Signal 23, Abu Dhabi, United Arab Emirates; sarah.abdulla@mbzuh.ac.ae; 4 Department of Physics, Khalifa University of Science and Technology, Abu Dhabi P.O. Box 127788, United Arab Emirates; shashikant.patole@ku.ac.ae

**Keywords:** β-cyclodextrin, silver nanoparticles, reactive oxygen species, hydrogen peroxide, creatinine

## Abstract

The β-cyclodextrin shell of synthesized silver nanoparticles (β***CD-AgNPs***) are found to enhance the detection of hydrogen peroxide in urine when compared to the Horse Radish Peroxidase assay kit. Nanoparticles are confirmed by the UV-Vis absorbance of their localized surface plasmonic resonance (LSPR) at 384 nm. The mean size of the β***CD-AgNPs*** is 53 nm/diameter; XRD analysis shows a face-centered cubic structure. The crystalline structure of type 4H hexagonal nature of the AgNPs with 2.4 nm β-CD coating onto is confirmed using aberration corrected high-resolution transmission electron microscopy (HRTEM). A silver atomic lattice at 2.50 Å and 2.41 Å corresponding to (100) and (101) Miller indices is confirmed using the HRTEM. The scope of β***CD-AgNPs*** to detect hydrogen peroxide (H_2_O_2_) in aqueous media and human urine is investigated. The test is optimized by examining the effect of volumes of nanoparticles, the pH of the medium, and the kinetic and temperature effect on H_2_O_2_ detection. The β***CD-AgNPs*** test is used as a refined protocol, which demonstrated improved sensitivity towards H_2_O_2_ in urine compared to the values obtained by the Horse Radish Assay kit. Direct assessment of H_2_O_2_ by the β***CD-AgNPs*** test presented always with a linear response in the nM, μM, and mM ranges with a limit of detection of 1.47 nM and a quantitation limit of 3.76 nM. While a linear response obtained from 1.3 to 37.3 nmoles of H_2_O_2_/mole creatinine with a slope of 0.0075 and regression coefficient of 0.9955 when the β***CD-AgNPs*** is used as refined test of creatinine. Values ranging from 34.62 ± 0.23 nmoles of H_2_O_2_/mole of creatinine and 54.61 ± 1.04 nmoles of H_2_O_2_/mole of creatinine when the matrix is not diluted and between 32.16 ± 0.42 nmoles of H_2_O_2_/mole of creatinine and 49.66 ± 0.80 nmoles of H_2_O_2_/mole of creatinine when the matrix is twice diluted are found in freshly voided urine of seven apparent healthy men aged between 20 and 40 years old.

## 1. Introduction

Reactive oxygen species (ROS) are very reactive unstable products of oxygen metabolism in all organisms, formed through different mechanisms. They are generated in mitochondria as result of energy production, or also as part of an antimicrobial or antiviral responses [1]. The process of generating ROS can be favored under exogenous stimuli such as environmental pollutants’ exposures, UV radiation, air pollution, and cigarette smoking [2]. ROS are used as biomarkers to gain information about the status of biological redox system, progression, and state of diseases and the status of health enhancement by enzymatic and non-enzymatic antioxidants in humans [3]. ROS are found to have a direct role in cell signaling namely: gene expression, apoptosis, and overall activation of cell signaling cataracts, and could serve as intra and intercellular messengers [4,5]. Mitochondrial electron transport process is found to be responsible for generating superoxide ($O_{2}^{•-}$) which is converted to hydrogen peroxide (H_2_O_2_) under the effect of superoxide dismutase or even under spontaneous dismutation [3]. In metal catalyzed reactions ROS are necessary intermediates that host electrons released by the oxidation reactions. Superoxide, for instance, is produced by electron transfer to O_2_ at Q_o_ site of ubiquinol-cytochrome c oxidoreductase, a complex formed from large number of polypeptides, three heme groups, and a Fe-S center [6]. Atomic oxygen has two lone pairs of electrons that occupy two different orbitals, which makes it more vulnerable to changes especially radicals’ formation, the consecutive acceptance of electrons by oxygen lead to the formation of many ROS species, namely: superoxide ($O_{2}^{•-}$); hydrogen peroxide ($H_{2}O_{2}$); hydroxyl radical (${\mathrm{HO}}^{•}$); and hydroxyl ion (${\mathrm{HO}}^{-}$). Other oxygen-containing ROS includes: alkoxy radicals (${\mathrm{RO}}^{•}$), nitric oxide (${\mathrm{NO}}^{•}$), peroxyl radical $({\mathrm{HOO}}^{•}$), sulfate radical (${\mathrm{SO}}_{4}^{•-}$), and lipids hydroperoxides (LOOH), all these ROS share the property of being able to cause oxidation of essential components of the cell [7]. The imbalance between the produced and removed ROS is referred to as “oxidative stress”. All survival organisms detoxify ROS through defense mechanisms that provide a balance between produced and removed ROS [8]. Oxidoreductase enzymes, such as superoxide dismutase catalyze the reaction of conversion of two superoxide molecules to one hydrogen peroxide molecule and one molecular oxygen [9]. Hydrogen peroxide is considered as important biomarker between other ROS due to superoxide’s dismutation by the superoxide dismutase (SOD) which acts as first line defense against oxidative stress [10]. In addition of being a major product of oxidase catalyzed reactions, H_2_O_2_ level is responsible for many cellular damages [11,12,13]. Sensing of H_2_O_2_ is significant in many fields, mainly medical and environmental, measuring the concentration of H_2_O_2_ in micro and nano levels is essential to control quantities of many important biomolecules in our diet such as carbohydrate, lipids, and proteins, and their monomeric molecules [14]. Many analytical assays have been used to quantify the concentration of H_2_O_2_ such as electrochemical [15,16,17,18], enzymatic [19,20,21], and colorimetric assays are still the most attractive due to their simplicity and cost effective [22,23,24,25,26]. The disadvantage of the analytical methods based on colorimetric detection of H_2_O_2_, is mainly due to their low sensitivity. A satisfactory reproducibility and high sensitivity for H_2_O_2_ detection is therefore difficult to obtain.

Recently there has been a lot of focus on metals nanoparticles characteristics for ROS detection methods [27,28]. This is due to their small size and extraordinary optical, magnetic, catalytic, and powered properties of nanoparticles that are not found in bulk materials [29,30]. Based on the hypothetical attributes of the nanomaterials such as nanoparticles, nanotubes and nanofibers these are used in the form of quantum dots (QDs) or noble metal nanoparticles (NPs); for instance, gold and silver for the development of chemical sensors and biosensors [31,32].

On the other hand, cyclodextrins are macromolecule oligosaccharides that occur naturally, and consist of six, seven, and eight glucose cycles that are joined by glycosidic bonds. They are created from enzymatic starch conversion [33]. Cyclodextrins are arranged like cones, and they can host small molecules, such as aromatic molecules and steroids inside their cavities. The interior cavity has a lipophilic character. However, the external surface of cyclodextrins is very soluble in water. This is what explain their highest solubility in water [34].

The encapsulation character of cyclodextrins lead to their wide use in drug delivery. β-cyclodextrin is occupying a middle position because of its moderated size cavity of 0.6 nm compared to α and γ-cyclodextrins [35]. Herein, a novel strategy based on mediated creatinine encapsulation by the β-cyclodextrin organic shell of the nanoparticles is developed. Due to the hosting ability of the β-cyclodextrin, the creatinine could easily be hosted by the shell and improves the stability and reproducibility of H_2_O_2_ concentration in urine. The test results are validated through comparison with previous assays and a commercially available assay kit.

## 2. Materials and Methods

### 2.1. Chemicals

All chemicals are purchased from Sigma Aldrich through a local company (Labcoltd-Dubai, UAE), they are used without any further purification as received unless otherwise stated. Soluble β-cyclodextrin (C_42_H_70_O_35_), sodium borohydride (NaBH_4_), hydrogen peroxide (H_2_O_2_, 35% *w/w*, d = 1.13 g/mL), buffer pH = 4.0 (0.1 M citric acid/sodium hydroxide), buffer pH = 7.0 (0.1 M potassium dihydrogen phosphate, disodium hydrogen phosphate, 12 hydrates), buffer pH = 10.0 (0.1 M borax/sodium hydrate), calcium nitrate tetrahydrate (Ca(NO_3_)_2_·4H_2_O, 99.95%), cobalt(II) nitrate hexahydrate (Co(NO_3_)_2_·6H_2_O, analytical grade), copper(II) sulfate pentahydrate (CuSO_4_·5H_2_O, analytical grade), Manganese(II) nitrate tetrahydrate (Mn(NO_3_)_2_·4H_2_O, analytical grade), nickel(II) nitrate (Ni(NO_3_)_2_, analytical grade), lead(II) nitrate (Pb(NO_3_)_2_, ACS reagent), zinc chloride (ZnCl_2_, ACS reagent) and sodium fluoride (NaF, ACS reagent, 99.99%) urea (CO(NH_2_)_2_, ACS reagent), D(+)-glucose (C_6_H_12_O_6_, anhydrous), ascorbic acid (C_6_H_8_O_6_, ≥99%) are purchased from Sigma Aldrich. Silver nitrate (AgNO_3_, 99.9+%) is purchased from Alfa Aesar, and ultrapure deionized water (from Millipore) is employed to formulate aqueous solutions. Hydrogen peroxide assay kits are acquired from Biomedical Scientific Services LLC, Al-Ain, UAE.

### 2.2. Preparation of β-CDAgNPs

β-cyclodextrin capped nanoparticles are prepared subsequent the modified procedure described by Yingju et al. [36]. In a 500 mL one neck round bottom flask, 200 mL of saturated solution of β-cyclodextrin (0.2% *w*/*v*) is stirred for a period of 30 min, then 3.0 mL of a 0.1 M solution of AgNO_3_ as source of Ag^+^ and 6.0 mL of 0.1 M of newly prepared NaBH_4_ are added to the mixture. A color change occurs instantly after the addition of NaBH_4_ from uncolored to greenish. This is a strong signal of the creation of silver nanoparticles. The solution is stirred for an additional time of 30 min. The produced nanoparticles are stable for a period of more than 6 months; nanoparticles are centrifuged to ×4000 rpm for 10 min preceding any utilization. The β-CDAgNPs are brought to analysis and afterward are used for the detecting of H_2_O_2_. Because the absorbance of the bare nanoparticles is very high, nanoparticles are diluted 3 times in all experiments.

### 2.3. Optimization of the H_2_O_2_ Detection by β-CDAgNPs

The detection of H_2_O_2_ by β-CDAgNPs is optimized using batch equilibrium experimentations at room temperature. Preliminary and final concentrations of H_2_O_2_ are assessed from Δabs = A_ref_ − A_sample_ (measured between the reference and the sample) using a UV-vis spectrophotometry. The effect of the volume of nanoparticles used is assessed as follow; in 9 test tubes, volumes of 0.75 mL of fixed concentration of H_2_O_2_ (40 mM), are reacted with volumes ranging from 0.30 to 2.70 mL of the nanoparticles’ solution. Experiments are completed at the pH of the suspension without modification (pHi). After achieving equilibrium, the solutions are analyzed using UV-vis spectrophotometry. While the effect of pH on the detection of H_2_O_2_ is assessed by adding H_2_O_2_ concentration spanning in the range of 5.0 to 50.0 mM, solutions are prepared from a 50 mM stock solution. To 10 test tubes containing each 0.75 mL of the nanoparticles’ solutions, 0.3 mL of H_2_O_2_ with concentration varying between 5.0 and 50.0 mM are added. Tubes are left to equilibrate for 3.0 min and then absorbances of samples and the reference (β***CD-AgNPs***) are recorded between 200 and 800 nm using the UV-vis spectrophotometry. Similar experiments are repeated in pH = 4.0 and pH = 10.0 using corresponding 0.1 M buffers to fix the pH. To study the effect of time (kinetic of the reaction), 0.3 mL of 40.0 mM H_2_O_2_ is prepared then added to 0.75 mL of β-CDAgNPs, absorbances are recorded at different timing from 0.0 to 10.0 min by UV-Vis spectrometry. The effect of temperature is studied to gain information about the spontaneity of the process. For this purpose, the kinetic of 0.3 mL of 40.0 mM H_2_O_2_ mixed with 0.75 mL of β-CDAgNPs is studied at three different temperatures of 25 °C, 37 °C, and 45 °C.

### 2.4. H_2_O_2_ Detection in Urine and Limit of Detection

The β***CD-AgNPs*** test is compared with a peroxidase assay test as reference. The peroxidase assay allows the development of a pink color when Horse Radish peroxidase (HRP) is mixed with the OxiRed probe and H_2_O_2_ sample. The working standards of peroxidase assay are made ranging from 1.0 to 10.0 nmoles of H_2_O_2_.

Working standards of the H_2_O_2_ detection using β***CD-AgNPs*** are made in three different ranges 4.7 to 32.0 nM, 4.7 to 32.0 μM and 4.7 to 32.0 mM by mixing varying volumes from 0.1 to 2.8 mL of their parent stock solution of 40.0 nM, 40.0 μM, and 40.0 mM with 0.75 mL of β***CD-AgNPs***. A corrected creatinine H_2_O_2_ test is made in the 1.33 to 37.3 nmoles of H_2_O_2_/mole of creatinine by mixing varied volumes of 0.1 to 2.8 mL of 40.0 nM H_2_O_2_ with 0.75 mL of β***CD-AgNPs*** in the presence of 300 µL of 1.0 mM creatinine.

Midstream urine samples were collected from seven healthy-looking men volunteers between 20 and 40 years in age. The collected samples were stored at 4 °C for a period of 12 h followed by centrifugation for 15 min at ×1000 rpm before use. H_2_O_2_ concentrations are measured in triplicates in the samples’ supernatants. To reduce the effect of the matrix, analysis of H_2_O_2_ was studied in three sets: the pure urine samples, twice and ten times diluted urine samples. Stock solutions and working standards of H_2_O_2_ are prepared from the 30% concentrated H_2_O_2_, from which 0.88 mM and 0.2 mM are gradually diluted using MilliQ ultra-pure water with a resistivity of 17.5 MΩ·cm (Fisherbrand Accu20 Ultrapure Water System, Loughborough, UK). Then, working standards ranging from 1.0 to 10.0 nmoles/microplate are freshly prepared from 0.2 mM using 0.1 M buffer solution supplied with the assay kit. The standards as well as urine samples were treated similarly as mentioned in Abcam Assay Kit’s procedure: 46 µL of 0.1 M buffer, 2 µL of OxiRed probe, and 2 µL of HRP are blended, forming a reaction mixture to which 50 µL of the standard or sample is added. The working standards and urine samples are incubated for 3 h, prior to absorbances recording versus the reaction mixture blank for the peroxidase assay kit at 570 nm. However, for the β***CD-AgNPs*** assay, 150 µL of the working standard or sample are mixed with 150 µL of the nanoparticles. Samples and working standards are incubated for 40 min, and absorbances are recorded at 384 nm against the 0.1 M buffer as a blank. The limit of detection (LOD) of β***CD-AgNPs*** assay test is verified using the standard deviation of the absorbance and slope of the calibration curve after the equation LOD = 3.3 σ/S [29], where σ is the standard deviation of three reading of the lowest possible concentration, made by mixing 0.75 mL of β***CD-AgNPs*** with 100 µL of H_2_O_2_ to give a total volume of 0.95 mL. The limit of quantitation (LOQ) is defined using the equation LOQ = 10 L_v_/S, where L_v_, is the lowest possible concentration identified by the test. S signifies the slope of the calibration curve in the nM range. Conversion between molar concentration and moles of H_2_O_2_ is made by multiplying the concentration by 300 μL, representing the volume of the microplate’s well. Measurements are made in triplicates and presented as Mean ± SD.

### 2.5. Instrumentation

The XRD analysis are made on a Bruker D8 Analytical diffractometer operated by the software DIFFRAC.SUITE at 40 kV and 40 mA, equipped with a copper anode and a graphite monochromator to select CuK_α1_ radiation (λ = 1.540 Å). Diffractions are recorded from 2θ = 5.0° to 80° with step increase of 0.03° and time of 0.5 s/step. To determine the abundance of elements in β***CD-AgNPs***, EDS is made concomitantly with SEM using a Tescan VEGA XM electron microscope machine provided with an X-Max 50 EDS detector’s Oxford Instruments (Tescan, Brno, Czech Republic), at 125 eV resolution and managed by the AZtecEnergy analysis software. The software VEGA TC is used to record SEM images of the β***CD-AgNPs*** using an accelerating voltage of 30 kV. TEM images are recorded by transferring the β***CD-AgNPs*** onto the TEM grid (Electron Microscopy Sciences 300 mesh grid made of a carbon layer deposited on copper). The aberration corrected TEM (Hillsboro, Oregon, USA, Thermo Fisher Scientific formerly FEI, Titan G2) is used for the structural characterization of the β***CD-AgNPs***, run at 300 kV and using spherical aberration)-correction. The hydrodynamic sizes of the nanoparticles are measured using the Nanotrac Wave II DLS analyzer (Microtrac, Pennsylvania, USA) working between 0.8 nm and 6.5 and is run for 15 cycles at a 90° angle and 26 °C.

## 3. Results and Discussion

**Figure 1:**
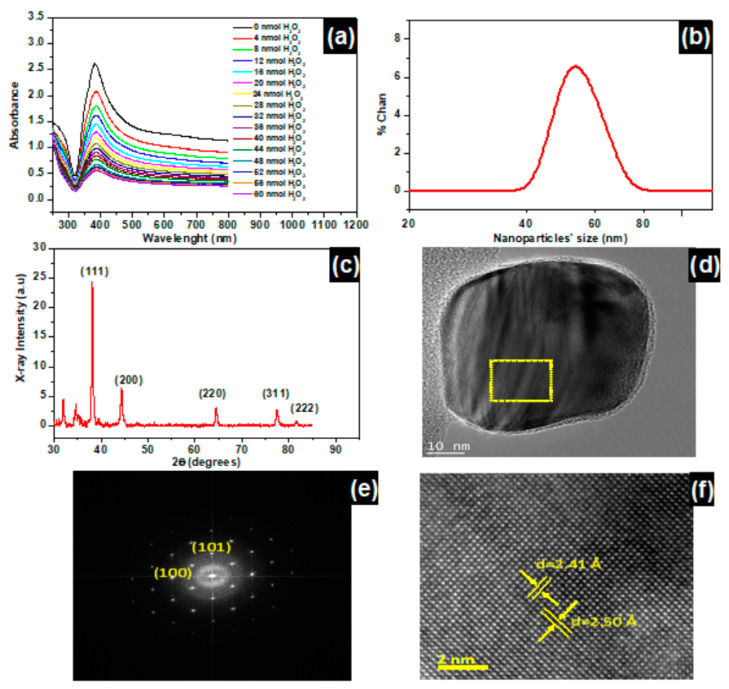
(**a**) βCD-AgNPs LSPR absorbances recorded at λ_max_ = 384 nm as function of H_2_O_2_ concentration increase, and nanoparticles diluted 3 times. (**b**) Size of βCD-AgNPs using dynamic Light scattering (DLS). (**c**) X-ray diffraction of βCD-AgNPs. (**d**) HRTEM micrograph of a typical 50 nm β***CD-AgNPs*** with 2.4 nm of βCD macromolecule coating (marked between two yellow lines in the image). (**e**) Fast Fourier Transform HRTEM image with Miller indices of the electron diffraction, indicating the crystalline structure of type 4H hexagonal nature of the nanoparticle. (**f**) HRTEM micrograph of chosen area marked with square in (**d**), showing that the interplanar distances are 2.50 Å and 2.41 Å corresponding to (100), (101) Miller indices.

Figure 1a represents the UV-Vis absorbance of the surface plasmon peaks of βCD-AgNPs with a characteristic peak at 384 nm. This peak is found to decrease as the concentration of H_2_O_2_ increases. The color of the nanoparticles faded from a green olive color to uncolored with the increase of the concentration of H_2_O_2_ (Figure S1, Supplementary Materials), suggesting a proportionality between the color change of the βCD-AgNPs and concentration change of H_2_O_2_. Consequently, color change is exploited as an optical test for the hydrogen peroxide detection.

The color change of the βCD-AgNPs, from green olive to uncolored, is accompanied by a gradual decrease in the absorption intensity of the characteristic plasmonic peak of the βCD-AgNPs at 384 nm due to atomic dissolution of βCD-AgNPs in the presence of H_2_O_2_, and this explanation is in accordance with the proposed mechanism by which the βCD-AgNPs are oxidized in the presence of H_2_O_2_ to release Ag^+^ to the medium. H_2_O_2_ may induce aggregation of the AgNPs, as is indicated by Wang et al. [37] during the regeneration of AuNPs from an aromatic thiol (ArSH) dissolved AuNPs solution. However, aggregation cannot be an overall mechanism of βCD-AgNPs interaction with H_2_O_2_ because the resulting solution does not have any absorbance.

The first parameter to check when optimizing a test is its selectivity, the βCD-AgNPs are mixed volume by volume with Ca^2+^, Co^2+^, Cu^2+^, Mn^2+^, Ni^2+^, Pb^2+^, Zn^2+^, F^−^, urea, glucose, and ascorbic acid as possible interfering species with H_2_O_2_. Little changes are assessed in the case of all interfering species, with the most pronounced change found for H_2_O_2_, results of this analysis are presented in Figure S2 (Supplementary Materials). Energy Disperse Spectroscopy (EDS) presented in Figure S3 (Supplementary Materials) demonstrates the presence of silver, carbon, and oxygen, and proves that β-cyclodextrin macromolecules successfully capped the silver metal. The weight percent of the silver is found to be the highest at 44.1 wt% followed by oxygen and carbon.

The βCD-AgNPs size distribution is determined using the dynamic light scattering (DLS) and the aberration-corrected high-resolution transmission electron microscopy (HRTEM) represented in Figure 1b,d (and Figure S4 in Supplementary Materials), respectively. The DLS size is found to be 52.0 nm/dimeter, however the HRTEM shows regular spherical shaped nanoparticles with average size varying between 19.4 and 53 nm/dimeter, and the high diameter presented by DLS has been attributed to the DLS measuring the hydrodynamic size of the particle which is the size of the βCD macromolecules shell and the liquid layer all around the particle. Shells may be involved in various forms of intermolecular forces (van der Waals forces and hydrogen bonding), while the HRTEM gives the actual size of the nanoparticle [38]. Zheng et al. demonstrated, however, that the hydrodynamic diameters of the nanoparticles can significantly vary with nanoparticles’ concentration and incident beam used in the instrument [39].

Figure 1c represents the XRD pattern of an oven dried βCD-AgNPs at 100 °C for a period of 24 h. The peaks at 38.12°, 44.35°, 64.60°, 77.33°, and 81.45° are correlated to the (111), (200), (220), (311), and (222) reticular planes of the crystalline Ag of the β***CD-AgNPs*** [40]. An enlargement of the βCD-AgNPs peaks in comparison to metallic silver (PDF no. 04–0783), is assigned to a nanosized Ag in the βCD-AgNPs material [41]. The Ag atomic lattice of the nanoparticles is also confirmed using HRTEM (Figure S5 in supplementary materials), Figure 1d presents a selected 50 nm βCD-AgNPs with 2.4 nm of βCD coating. Fast Fourier Transform HRTEM image with Miller indices of the electron diffraction is shown in Figure 1e. The image clearly shows the crystalline structure of type 4H hexagonal nature of the nanoparticle. The interplanar distances in HRTEM are 2.50 Å and 2.41 Å correspond to (100), (101) Miller indices [42].

**Figure 2:**
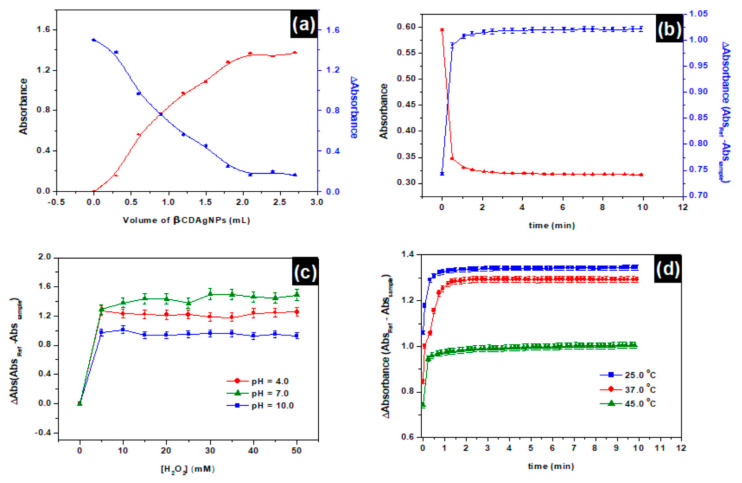
(**a**) Effect of β***CD-AgNPs*** volume on the detection of H_2_O_2_, Experiment condition [H_2_O_2_] = 40 mM, equilibrium time (3 min), pHi = 9.28, T = 22.5 °C. (**b**) Impact of time (kinetic) of H_2_O_2_ recognition by β***CD-AgNPs*** as a function of equilibration time at 25 °C; [H_2_O_2_]_i_ = 40 mM; V (AgNPs)= 0.75 mL; pHi (β***CD-AgNPs***) ~ 9.28; (**c**) Effect of different pH on H_2_O_2_ by β***CD-AgNPs***. Experiment condition [H_2_O_2_] = from 1.0 to 50.0 mM increasing with a step of 5.0 mM, equilibration time (3 min), T = 25 °C. (**d**) Kinetic of the effect of temperature on H_2_O_2_ detection by β***CD-AgNPs***; V(β***CD-AgNPs***) = 0.75 mL; pH = 9.28; [H_2_O_2_]_i_ = 40.0 mM; Absorbances in all experiments are recorded at LSPR λ_max_ = 384 nm and nanoparticles were diluted 3 times.

Figure 2a represents the evolution of absorbances of different samples at different volumes of βCD-AgNPs. An increase in the concentration of βCD-AgNPs (volume increase) is faced with a decrease in the sample’s absorbance, ΔAbs represents the difference between the reference unreacted βCD-AgNPs minus the absorbance of the sample, which is found to increase with concentration increase of H_2_O_2_. Even though the volume increase of βCD-AgNPs nanoparticles is expected to increase the number of active sites of interaction with H_2_O_2_, however, the number of H_2_O_2_ molecules circumvent the sites of interaction’s number provided by the material. At 2.0 mL of βCD-AgNPs, enough active sites of interactions have been provided by the material to interact in a mole-to-mole ratio with the H_2_O_2_ in the medium. After this point, the number of active sites provided exceed the required number and a saturation is observed on words. The kinetic of H_2_O_2_ interaction with βCD-AgNPs is presented in Figure 2b, it can be observed that increasing the time of the reaction lead to direct increase in the ΔAbs until it reaches a plateau. In the beginning, the material has many sites of interaction, with time increase these sites are occupied by H_2_O_2_, while the time proceeding these sites are fully occupied by H_2_O_2_ and therefore saturation is achieved. The kinetic of the reaction is found to be very fast, equilibrium is attained at time less than 0.5 min, the sites of interaction of βCD-AgNPs almost consumed after 0.5 min to attain equilibrium.

Figure 2c shows an increase in ∆Abs by increasing the concentration of H_2_O_2_ until ∆Abs reaches a plateau for the three pH (pH = 4.0, pH = 7.0, and pH = 10.0). The ΔAbs is lower in alkaline pH compared to neutral pH = 7.0 and acidic pH = 4.0, lower ∆Abs corresponds to a higher Abs, which is indicative that the nanoparticles are more stable in alkaline mediums, this behavior could be explained based on hydroxide anions could enhance aggregation of the nanoparticles. The acidic pH = 4 is still presenting a ΔAbs lower (high Abs) than the neutral pH = 7.0 which may be explained by competition reaction between the hydronium ion and H_2_O_2_. From Figure 2d, it can be concluded that β***CD-AgNPs*** operate very well at high temperature, since 45 °C is presented with lowest ∆Abs (highest Abs). Thirty-seven degrees, the physiological temperature where most of the biological samples are extracted, presented with the second lowest ∆Abs when adopted for biological analysis. The temperature increases the speed of collision between the H_2_O_2_ and sites of interactions on the β***CD-AgNPs***. Increasing the temperature slows down the process. The reaction is therefore classified as exothermic.

**Figure 3:**
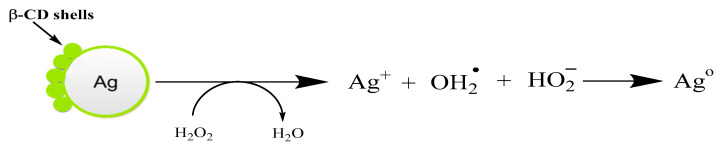
Schematic representation of the mechanism of βCD-AgNPs reaction with hydrogen peroxide.

The mechanism of interaction of βCD-AgNPs, with H_2_O_2_ represented in Figure 3, is based on the oxidative dissolution of the nanoparticles to release Ag^+^ and other reactive oxygen species such as OH^•^ which will further dissolute the nanoparticles. Molleman and Hiemstra [43] demonstrated that the shape of the AgNPs plays a big role in their reactivity, and they found that nanorods and nanoprisms are more reactive than nanospheres because they exhibit exposed facets and therefore react faster.

**Figure 4:**
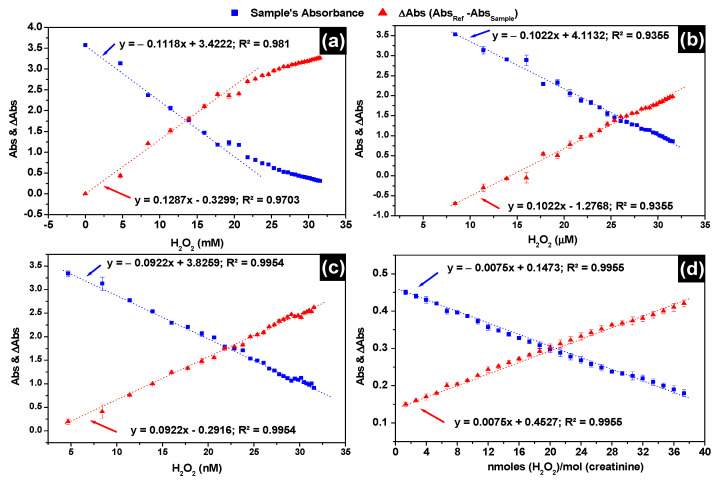
Calibration curves of H_2_O_2_ detection using LSPR absorbances of the β***CD-AgNPs*** and LSPR ΔAbsorbance between the reference and the sample (**a**) 4.7 to 32.0 mM H_2_O_2_ range; (**b**) 4.7 to 32.0 µM H_2_O_2_ range; (**c**) 4.7 to 32.0 nM H_2_O_2_ range; (**d**) 1.3 to 37.3 nmoles of H_2_O_2_/mole of creatinine range in the presence of 10 mM creatinine. Absorbances in all experiments are recorded at LSPR λ_max_ = 384 nm and nanoparticles were diluted 3 times.

To measure the level of H_2_O_2_ in patients’ urine, calibration curves are established in mM, μM and nM concentration ranges, which are presented in Figure 4a–c, respectively. Markedly, the β***CD-AgNPs*** test presented with a large linear range from 5.0 nM through the whole μM range up to 17.7 mM concentration (Figure 4a) where the deviation from linearity start showing. In Figure 4d, this is represented by a refined protocol of H_2_O_2_ response in the presence of 10 mM of creatinine. The sensitivity is almost 12-folds lower, using the refined protocol with linearity maintained from 1.3 up to 37.3 nmoles of H_2_O_2_/mole of creatinine. This decrease in sensitivity is due to the encapsulation creatinine with β-CD shell of the nanoparticles. β-CD is well known to form 1:1 complexes with neutral guest species, in solution, the nonpolar cavity of β-CD is occupied with water molecules which is not favorable energetically. Water molecules are substituted with appropriate guest molecules such as creatinine that are less polar than water molecule. Limit of H_2_O_2_ detection using β***CD-AgNPs*** is determined in the nM calibration curve applying the equation LOD = 3σ/S, where σ is the standard deviation of the response of three urine samples replicates at wavelength of 384 nm. The limit of detection is found to be 1.47 nM when Absorbance of β***CD-AgNPs*** is used directly to assess H_2_O_2_, while 0.041 nM is found when ΔAbs is used instead. The detection limit is very low compared to other H_2_O_2_ sensors reported in Table 1. The limit of quantitation (LOQ) is found from the formula: LOQ = 10(S_y_/S), where S_y_ and S are the slope and standard deviation of the response obtained from the calibration of the test. A LOQ of 3.76 nM is found for the β***CD-AgNPs*** test when Abs is used and 0.14 nM when ΔAbs is used, indicating that ΔAbs always yield lower LOD and LOQ compared to Abs.

**Figure 5:**
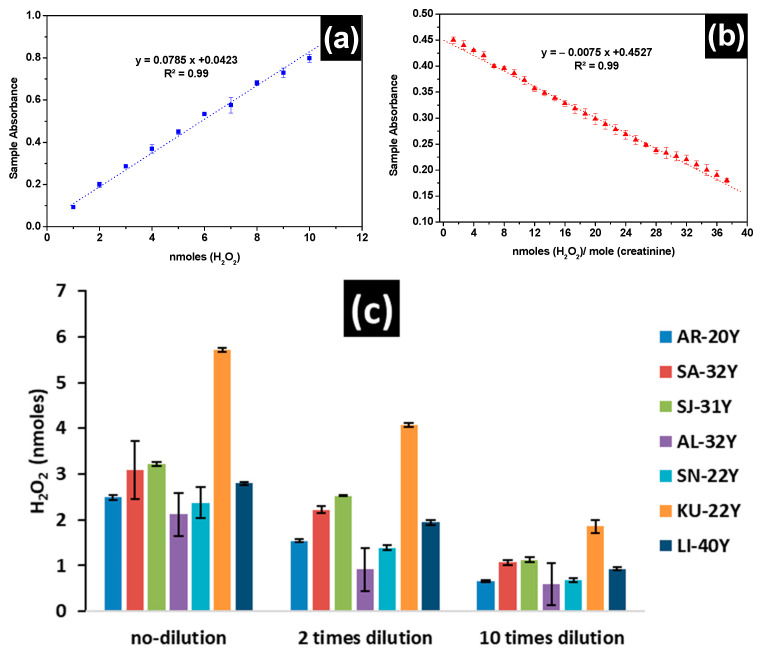

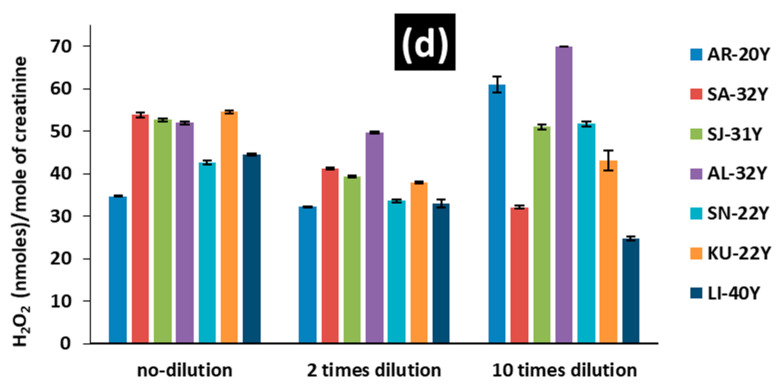
Calibration curves used for the measurement of H_2_O_2_ levels in 7 patients’ urine samples (**a**) using horse radish commercial assay kit, (**b**) using LSPR sample absorbance when mixed with β***CD-AgNPs*** in the presence of 10 mM creatinine, and (**c**,**d**) H_2_O_2_ levels of freshly voided midstream urine of the 7 healthy participants (all men) aged between 20 and 40 years old, using the commercial assay kit and β***CD-AgNPs***, respectively.

In Figure 5a,b the calibration curves used to assess the levels of H_2_O_2_ in freshly voided midstream urine of the 7 healthy men aged between 20 and 40 years are presented, using the assay kit and the LSPR of β***CD-AgNPs***, respectively. Linear responses are obtained for both tests. For the assay kit a linear response was in the range of 1.0 nmoles to 10.0 nmoles (10–100 μM) with a slope of 0.0785 and a regression coefficient of 0.995, while a linear response 1.33 to 37.3 nmoles of H_2_O_2_/moles of creatinine with a slope of 0.0075 and regression coefficient of 0.9955 when the β***CD-AgNPs*** test is used in the presence of 10.0 mM of creatinine. The concentration of H_2_O_2_ is dependent on the dilution for all participants, except KU-22, all participants showed lower concentration of H_2_O_2_ when samples are diluted 10 times and the assay kit is used (Figure 5c). The average concentration of H_2_O_2_ in urine using the assay kit is found to vary between 2.11 ± 0.048 nmoles (21.1 ± 0.48 μM) and 3.22 ± 0.046 nmoles (32.2 ± 0.46 μM) when the matrix is not diluted, between 1.38 ± 0.042 nmoles (13.8 ± 0.42 μM) and 2.52 ± 0.019 nmoles (25.2 ± 0.19 μM) when the matrix is diluted twice and between 0.60 ± 0.051 nmoles (6.0 ± 0.51 μM) and 1.12 ± 0.050 nmoles (12.1 ± 0.50 μM) when the matrix is diluted 10 times. In this current study, the H_2_O_2_ total concentration in freshly voided urine in the 7 healthy subjects using the assay kit are comparable to values reported by Long et al. [52] who found values between 11 and 173 μM when analyzing freshly secreted urine of subjects aged from 19 to 38 years old using oxoglutarate decarboxylation assay. H_2_O_2_ levels of 17.28 μM are also reported in normal participants not suffering from any disease [53]. However, very high concentrations of H_2_O_2_ were noticed in ill subjects, 89.10 ± 8.73, 85.37 ± 15.45, 91.35 ± 17.79, and 68.02 ± 7.23 μM are reported in subjects suffering from helminth + protozoa, protozoa only, helminth and intestinal parasitic, respectively. Lower levels of H_2_O_2_ in the order of 0.84 to 5.71 µM are also reported in fasting subjects [54].

The detection and reproducibility of H_2_O_2_ in urine is enhanced when the creatinine corrected test is used instead of direct test for H_2_O_2_, because of the probable interaction between creatinine and H_2_O_2_. Even though no clear reaction has been documented in the literature, Hanif et al. [55] proposed a mechanism for the interaction of creatinine with H_2_O_2_ in the presence of a catalyst such as Co^2+^ which generates a tetrahedral spirodioxetane intermediates. The unstable spirodioxetane intermediates decompose and results in the formation of an excited-state species with concomitant elimination of CO_2_.

In terms of creatinine corrected H_2_O_2_ levels in urine, β***CD-AgNPs*** test shows values ranging from 34.62 ± 0.23 nmoles of H_2_O_2_/mole of creatinine and 54.61 ± 1.04 nmoles of H_2_O_2_/mole of creatinine when the matrix is not diluted and between 32.16 ± 0.42 nmoles of H_2_O_2_/mole of creatinine and 49.66 ± 0.80 nmoles of H_2_O_2_/mole of creatinine when the matrix is twice diluted. When the matrix is diluted 10 times, the level of H_2_O_2_ is not consistent, yet it increases for AR-20, AL-32, and SN-22, while decreasing for SA-32, SJ-31, KU-22, and Li-40. This lack of consistency in the test results at 10-times dilution could be attributed to the looseness of the nanoparticles LSPR at high dilutions. Minimal data are available in the literature about creatinine corrected H_2_O_2_ concentration. Yuen et al. [56], for instance, reported values between 90–1164 μmoles H_2_O_2_/mole creatinine using a refined protocol taking into account the presence of creatinine in the H_2_O_2_ matrix that increases the concentration of H_2_O_2_. These values are significantly higher than values found for β***CD-AgNPs*** test and the variations are mainly due to the stability and biological variations of the sample which limit the use of hydrogen peroxide in urine as a potential biomarker of whole-body oxidative stress.

## 4. Conclusions

β***CD-AgNPs*** have been successfully synthesized, and the synthesized NPs are characterized by UV−visible, DLS, EDX, XRD, and HRTEM analysis. The assay using βCD acted as a shell to cap and stabilize silver nanoparticles. Furthermore, a simple and instant colorimetric assay for H_2_O_2_ detection in the commercial oxidant solution is established via synthesized β***CD-AgNPs***. The H_2_O_2_ detection optimum conditions were studied in detail. An increase in the volume of nanoparticles is faced with a decrease in the LSPR absorbance of the suspended nanoparticles mixed with 40.0 mM H_2_O_2_ concentration. The optimum pH is found to be equal to 10.0. The kinetic of the reaction is found to be fast, and equilibrium is established between the nanoparticles and H_2_O_2_ in the first 0.5 min. The thermodynamics of the equilibrium show that the process is exothermic, and the reaction is favored at 37 °C compared to 25 °C. The β-cyclodextrin organic shell of the synthesized silver nanoparticles (β***CD-AgNPs***) are found to enhance the detection of hydrogen peroxide in urine through encapsulation mediated process of creatinine when compared to the commercial assay kit. Surely the assay kit gives reproducible results of total H_2_O_2_ in urine, values varying between 0.60 ± 0.051 nmoles (6.0 ± 0.51 μM) and 3.22 ± 0.046 nmoles (32.2 ± 0.46 μM) are found at different dilution rates, suggesting that the test is affected by the matrix complexity. β***CD-AgNPs*** test provided an optical creatinine corrected test with high selectivity and sensitivity for H_2_O_2_, with values ranging from 24.82 ± 1.50 nmoles of H_2_O_2_/mole of creatinine and 70.0 ± 1.57 nmoles of H_2_O_2_/mole of creatinine. The test is also affected by the complexity of the matrix. These values are lower than creatinine corrected H_2_O_2_ reported in the literature (90–1164 μmoles H_2_O_2_/mole creatinine), and the variations may be due to the stability of the samples and limit the use of hydrogen peroxide in urine as a potential biomarker of whole-body oxidative stress.

## Figures and Tables

**Table 1:** Analytical performance of the β***CD-AgNPs*** during H_2_O_2_ detection in comparison with other H_2_O_2_ non-enzymatic tests.

Method	Limit of Detection (LOD)	Linear Range	Reference
Cu/Electrochemically reduced graphene oxide	1.87 nM	0.01–1 mM	[44]
CoHCF Nanoparticles	9.31 μM	8–7500 μM	[45]
Co-NC/NF	10 μM	0–5.0 mM	[28]
GC/ILC/(CNT-Fe-Ni)	0.971 nM	0.007–1000 μM	[46]
N, S co-doped G/CNT-Fe3C	272 nM	0.002–10.37 mM	[47]
Ti/ML-TNT/HRP/Nafion	12 nM	41.2 nM–6.0 μM	[48]
CuCo_2_O_4_/rGO	80 nM	30–5010 μM	[49]
Brown Algae AgNPs	8.6 nM	1–120 μM	[50]
Algae-AgNPs	1.77 nM	4.70–32.0 nM	[51]
Abs of β***CD-AgNPs***	1.47 nM	5.0–31.0 nM	This work
ΔAbs of β***CD-AgNPs***	0.041 nM		

## Data Availability

Not applicable.

## Supplementary Materials

The following are available online at https://www.mdpi.com/article/10.3390/nano11081897/s1, Figure S1: Photographs of β***CD-AgNPs*** exposed to increasing concentrations of H_2_O_2_ from the right to the left, Figure S2: LSPR absorbances of β***CD-AgNPs*** reacted with potential interfering species equilibrium time = 3.0 min; pH_0_ = 7.0, T = 25 °C, Figure S3: (a) Scanning Electron microscopy (SEM) and (b) Energy Disperse Spectroscopy (EDS) of β***CD-AgNPs***, Figure S4: (a,b) TEM micrograph of a typical β***CD-AgNPs***. The dotted rectangle indicates the region of interest (ROI) used for the analysis. (c) A 2.4 nm βCD macromolecule coating is indicated by the lines and arrows in the figure. The intensity profile along the dotted line in (c) is shown in (d) indicating 2.4 nm thickness. The ROI and profile are realized using the Gatan digital micrograph platform, Figure S5: TEM micrograph of β***CD-AgNPs***, with (a) 1.0 μm, (b) 50 nm, (c) 100 nm, and (d) 200 nm size scale bars taken at different magnification.

## References

[1] Murphy M.P. (2009). How mitochondria produce reactive oxygen species. Biochem. J..

[2] Boukhenouna S., Wilson M.A., Bahmed K., Kosmider B. (2018). Reactive oxygen species in chronic obstructive pulmonary disease. Oxid. Med. Cell. Longev..

[3] Marrocco I., Altieri F., Peluso I. (2017). Measurement and clinical significance of biomarkers of oxidative stress in humans. Oxid. Med. Cell. Longev..

[4] Ma J., Zhao D., Lu H., Huang W., Yu D. (2017). Apoptosis Signal-Regulating Kinase 1 (ASK1) Activation is Involved in Silver Nanoparticles Induced Apoptosis of A549 Lung Cancer Cell Line. J. Biomed. Nanotechnol..

[5] Endo T., Yanagida Y., Hatsuzawa T. (2008). Quantitative determination of hydrogen peroxide using polymer coated Ag nanoparticles. Measurement.

[6] Bae Y.S., Oh H., Rhee S.G., Do Yoo Y. (2011). Regulation of reactive oxygen species generation in cell signaling. Mol. Cells.

[7] Zorov D.B., Juhaszova M., Sollott S.J. (2014). Mitochondrial reactive oxygen species (ROS) and ROS-induced ROS release. Physiol. Rev..

[8] Rezayian M., Niknam V., Ebrahimzadeh H. (2019). Oxidative damage and antioxidative system in algae. Toxicol. Rep..

[9] Romero-Puertas M.C., Corpas F.J., Sandalio L.M., Leterrier M., Rodríguez-Serrano M., Del Río L.A., Palma J.M. (2006). Glutathione reductase from pea leaves: Response to abiotic stress and characterization of the peroxisomal isozyme. New Phytol..

[10] Mallick N., Mohn F.H. (2000). Reactive oxygen species: Response of algal cells. J. Plant Physiol..

[11] Zorov D.B., Plotnikov E.Y., Jankauskas S.S., Isaev N.K., Silachev D.N., Zorova L.D., Pevzner I.B., Pulkova N.V., Zorov S.D., Morosanova M.A. (2012). The phenoptosis problem: What is causing the death of an organism? Lessons from acute kidney injury. Biochemistry.

[12] Kozlov A.V., Bahrami S., Calzia E., Dungel P., Gille L., Kuznetsov A.V., Troppmair J. (2011). Mitochondrial dysfunction and biogenesis: Do ICU patients die from mitochondrial failure?. Ann. Intensive Care.

[13] de Bie M.K., Buiten M.S., Rabelink T.J., Jukema J.W. (2012). How to reduce sudden cardiac death in patients with renal failure. Heart.

[14] Gaikwad R., Thangaraj P.R., Sen A.K. (2021). Direct and rapid measurement of hydrogen peroxide in human blood using a microfluidic device. Sci. Rep..

[15] Zong L., Ruan L., Li J., Marks R.S., Wang J., Cosnier S., Zhang X., Shan D. (2021). Fe-MOGs-based enzyme mimetic and its mediated electrochemiluminescence for in situ detection of H_2_O_2_ released from Hela cells. Biosens. Bioelectron..

[16] Yin T., Wang H., Li J., Yuan B., Qin W. (2021). Translating potentiometric detection into non-enzymatic amperometric measurement of H_2_O_2_. Talanta.

[17] Fiore L., Mazzaracchio V., Galloni P., Sabuzi F., Pezzola S., Matteucci G., Moscone D., Arduini F. (2021). A paper-based electrochemical sensor for H2O2 detection in aerosol phase: Measure of H_2_O_2_ nebulized by a reconverted ultrasonic aroma diffuser as a case of study. Microchem. J..

[18] Tao N., Xu Y., Wang L., Yang W., Liu Y. (2021). Hollow porous N-doped carbon-based Co4N with peroxidase-like activity for detection of H_2_O_2_ under non-physiologic conditions. Microchem. J..

[19] Atlante A., Passarella S. (1999). Detection of reactive oxygen species in primary cultures of cerebellar granule cells. Brain Res. Protoc..

[20] Gomes A., Fernandes E., Lima J.L. (2005). Fluorescence probes used for detection of reactive oxygen species. J. Biochem. Biophys. Methods.

[21] Poulsen A.K., Scharff-Poulsen A.M., Olsen L.F. (2007). Horseradish peroxidase embedded in polyacrylamide nanoparticles enables optical detection of reactive oxygen species. Anal. Biochem..

[22] Wang Y., Liu X., Wang M., Wang X., Ma W., Li J. (2021). Facile synthesis of CDs@ZIF-8 nanocomposites as excellent peroxidase mimics for colorimetric detection of H_2_O_2_ and glutathione. Sens. Actuators B Chem..

[23] Guo T., Xu T., Xia W., Carrier A.J., Wang L., Zhang X. (2021). Graphene oxide and CuO double quantum dot composites (GOQD-q-CuO) with enhanced haloperoxidase-like activity and its application in colorimetric detection of H_2_O_2_ and glucose. Mater. Chem. Phys..

[24] Chen P., Zhong H., Li X., Li M., Zhou S. (2021). Palygorskite@Co_3_O_4_ nanocomposites as efficient peroxidase mimics for colorimetric detection of H_2_O_2_ and ascorbic acid. Appl. Clay Sci..

[25] Remani K.C., Binitha N.N. (2021). Cobalt doped ceria catalysts for the oxidative abatement of gaseous pollutants and colorimetric detection of H_2_O_2_. Mater. Res. Bull..

[26] Chi K., Guan Y., Zhang X., Yang T., Meng S., Hu R., Yang Y. (2021). Iodide/metal-organic frameworks (MOF) -mediated signal amplification strategy for the colorimetric detection of H_2_O_2_, Cr_2_O_7_^2−^ and H_2_S. Anal. Chim. Acta.

[27] Xu X., Luo P., Yang H., Pan S., Liu H., Hu X. (2021). Regulating the enzymatic activities of metal-ATP nanoparticles by metal doping and their application for H_2_O_2_ detection. Sens. Actuators B Chem..

[28] Riaz M.A., Yuan Z., Mahmood A., Liu F., Sui X., Chen J., Huang Q., Liao X., Wei L., Chen Y. (2020). Hierarchically porous carbon nanofibers embedded with cobalt nanoparticles for efficient H_2_O_2_ detection on multiple sensor platforms. Sens. Actuators B Chem..

[29] Elgamouz A., Bajou K., Hafez B., Nassab C., Behi A., Haija M.A., Patole S.P. (2020). Optical Sensing of Hydrogen Peroxide Using Starch Capped Silver Nanoparticles, Synthesis, Optimization and Detection in Urine. Sens. Actuators Rep..

[30] Ju-Nam Y., Lead J.R. (2008). Manufactured nanoparticles: An overview of their chemistry, interactions and potential environmental implications. Sci. Total Environ..

[31] Angioletti-Uberti S. (2017). Theory, simulations and the design of functionalized nanoparticles for biomedical applications: A Soft Matter Perspective. NPJ Comput. Mater..

[32] Yang D., Chen Y., Peng H., Chen G., Lin Z. (2018). An integrated experimental and theoretical study on the optical properties of uniform hairy noble metal nanoparticles. Nanoscale.

[33] Jansook P., Ogawa N., Loftsson T. (2018). Cyclodextrins: Structure, physicochemical properties and pharmaceutical applications. Int. J. Pharm..

[34] Szejtli J. (1998). Introduction and general overview of cyclodextrin chemistry. Chem. Rev..

[35] Dodero A., Schlatter G., Hébraud A., Vicini S., Castellano M. (2021). Polymer-free cyclodextrin and natural polymer-cyclodextrin electrospun nanofibers: A comprehensive review on current applications and future perspectives. Carbohydr. Polym..

[36] Fan Y., Liu Z., Zhan J. (2009). Synthesis of starch-stabilized Ag nanoparticles and Hg 2 recognition in aqueous media. Nanoscale Res. Lett..

[37] Wang F., He C., Han M., Wu J.H., Xu G.Q. (2012). Chemical controlled reversible gold nanoparticles dissolution and reconstruction at room-temperature. Chem. Commun..

[38] Patil R.B., Chougale A.D. (2021). Analytical methods for the identification and characterization of silver nanoparticles: A brief review. Mater. Today Proc..

[39] Zheng T., Bott S., Huo Q. (2016). Techniques for accurate sizing of gold nanoparticles using dynamic light scattering with particular application to chemical and biological sensing based on aggregate formation. ACS Appl. Mater. Interfaces.

[40] Shameli K., Ahmad M.B., Jazayeri S.D., Shabanzadeh P., Sangpour P., Jahangirian H., Gharayebi Y. (2012). Investigation of antibacterial properties silver nanoparticles prepared via green method. Chem. Cent. J..

[41] Zargar M., Hamid A.A., Bakar F.A., Shamsudin M.N., Shameli K., Jahanshiri F., Farahani F. (2011). Green synthesis and antibacterial effect of silver nanoparticles using *Vitex negundo* L.. Molecules.

[42] Virgen-Ortiz A., Limón-Miranda S., Soto-Covarrubias M.A., Apolinar-Iribe A., Rodríguez-León E., Iñiguez-Palomares R. (2015). Biocompatible silver nanoparticles synthesized using rumex hymenosepalus extract decreases fasting glucose levels in diabetic rats. Dig. J. Nanomater. Biostruct..

[43] Molleman B., Hiemstra T. (2015). Surface Structure of Silver Nanoparticles as a Model for Understanding the Oxidative Dissolution of Silver Ions. Langmuir.

[44] Temur E., Eryiğit M., Kurt Urhan B., Demir Ü., Öznülüer Özer T. (2021). Cu/Electrochemically reduced graphene oxide layered nanocomposite for non-enzymatic H_2_O_2_ sensor. Mater. Today Proc..

[45] Banavath R., Srivastava R., Bhargava P. (2021). Improved non-enzymatic H2O2 sensors using highly electroactive cobalt hexacyanoferrate nanostructures prepared through EDTA chelation route. Mater. Chem. Phys..

[46] Atta N.F., Abdel Gawad S.A., Galal A., Razik A.A., El-Gohary A.R.M. (2021). Efficient electrochemical sensor for determination of H_2_O_2_ in human serum based on nano iron-nickel alloy/carbon nanotubes/ionic liquid crystal composite. J. Electroanal. Chem..

[47] Karuppiah C., Venkatesh K., Arunachalam P., Ramaraj S.K., Al-Mayouf A.M., Yang C. (2021). Optimization of S-dopant on N, S co-doped graphene/CNT-Fe3C nanocomposite electrode for non-enzymatic H_2_O_2_ sensor. Mater. Lett..

[48] Chithra Lekha P., Ram Babu Y., Fidal Kumar V.T., Chandra T.S., Roy S.C. (2021). Investigation of Photo-induced Enhancement of Sensitivity and Electrochemical Surface Phenomenon of Multileg TiO_2_ Sensor Device towards H_2_O_2_. J. Electroanal. Chem..

[49] Jiang L., Zhao Y., Zhao P., Zhou S., Ji Z., Huo D., Zhong D., Hou C. (2021). Electrochemical sensor based on reduced graphene oxide supported dumbbell-shaped CuCo_2_O_4_ for real-time monitoring of H_2_O_2_ released from cells. Microchem. J..

[50] Farrokhnia M., Karimi S., Momeni S., Khalililaghab S. (2017). Colorimetric sensor assay for detection of hydrogen peroxide using green synthesis of silver chloride nanoparticles: Experimental and theoretical evidence. Sens. Actuators B Chem..

[51] Elgamouz A., Idriss H., Nassab C., Bihi A., Bajou K., Hasan K., Abu Haija M., Patole S.P. (2020). Green Synthesis, Characterization, Antimicrobial, Anti-Cancer, and Optimization of Colorimetric Sensing of Hydrogen Peroxide of Algae Extract Capped Silver Nanoparticles. Nanomaterials.

[52] Long L.H., Evans P.J., Halliwell B. (1999). Hydrogen peroxide in human urine: Implications for antioxidant defense and redox regulation. Biochem. Biophys. Res. Commun..

[53] Chandramathi S., Suresh K., Anita Z.B., Kuppusamy U.R. (2009). Elevated levels of urinary hydrogen peroxide, advanced oxidative protein product (AOPP) and malondialdehyde in humans infected with intestinal parasites. Parasitology.

[54] Varma S.D. (1989). Radio-isotopic determination of subnanomolar amounts of peroxide. Free Radic. Res. Commun..

[55] Hanif S., John P., Gao W., Saqib M., Qi L., Xu G. (2016). Chemiluminescence of creatinine/H_2_O_2_/Co^2+^ and its application for selective creatinine detection. Biosens. Bioelectron..

[56] Yuen J., Benzie I. (2003). Hydrogen peroxide in urine as a potential biomarker of whole body oxidative stress. Free Radic. Res..

