# Supplementary material for: Encapsulation Capacity of β-Cyclodextrin Stabilized Silver Nanoparticles towards Creatinine Enhances the Colorimetric Sensing of Hydrogen Peroxide in Urine
Source: Nanomaterials (Basel). 2021 Jul 24;11(8):1897. doi: 10.3390/nano11081897 (PMC8399024; doi:10.3390/nano11081897)
Supplement: Supplementary file 1 [file nanomaterials-11-01897-s001.zip › nanomaterials-1275736-supplementary.pdf]

nanomaterials

Article

Encapsulation Capacity of β-Cyclodextrin Stabilized Silver Nanoparticles towards Creatinine Enhances the Colorimetric Sensing of Hydrogen Peroxide in Urine

Abdelaziz Elgamouz [1,*], Chahlaa Nassab [1], Alaa Bihi [1], Somaya A. I. Mohamad [1], Aisha H. S. A. Almusafri [1], Salman S. Alharthi [2], Sarah A. E. Abdulla [3] and Shashikant P. Patole [4]

[1] Department of Chemistry, College of Sciences, University of Sharjah, Sharjah P.O. Box 27272, United Arab Emirates; U19103696@sharjah.ac.ae (C.N.); abihi@sharjah.ac.ae (A.B.); u00041765@sharjah.ac.ae (S.A.I.M.); u00042707@sharjah.ac.ae (A.H.S.A.A.)
[2] Department of Chemistry, College of Science, Taif University, P.O. Box 11099, Taif 21944, Saudi Arabia; s.a.alharthi@tu.edu.sa
[3] Mohamed Bin Zayed University for Humanities, Al Muroor Street, Signal 23, Abu Dhabi, United Arab Emirates; sarah.abdulla@mbzuh.ac.ae
[4] Department of Physics, Khalifa University of Science and Technology, Abu Dhabi P.O. Box 127788, United Arab Emirates; shashikant.patole@ku.ac.ae
* Correspondence: aelgamouz@sharjah.ac.ae; Tel.: +971-65166769

Citation: Elgamouz, A.; Nassab, C.; Bihi, A.; Somaya A. I. Mohamad; Aisha H. S. A. Almusafri; Alharthi, S.S.; Sarah A. E. Abdulla; Patole, S.P. Encapsulation Capacity of β-Cyclodextrin Stabilized Silver Nanoparticles towards Creatinine Enhances the Colorimetric Sensing of Hydrogen Peroxide in Urine. *Nanomaterials* **2021**, *11*, 1897. https://doi.org/10.3390/nano11081897

Academic Editors: Noel Rodriguez, Diego P. Morales and Almudena Rivad-eneyra

Received: 10 June 2021
Accepted: 20 July 2021
Published: 24 July 2021

Publisher's Note: MDPI stays neutral with regard to jurisdictional claims in published maps and institutional affiliations.

Copyright: © 2021 by the authors. Submitted for possible open access publication under the terms and conditions of the Creative Commons Attribution (CC BY) license (http://creativecommons.org/licenses/by/4.0/).

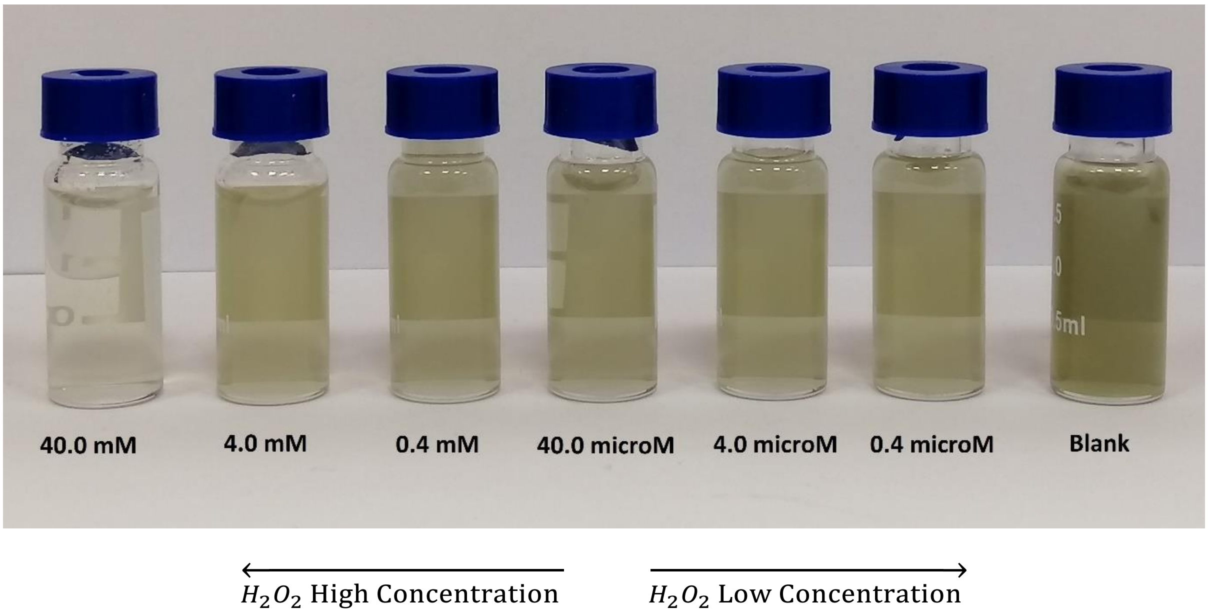

Figure S1. Photographs of β-CD-AgNPs exposed to increasing concentrations of H_2O_2 from the right to the left.

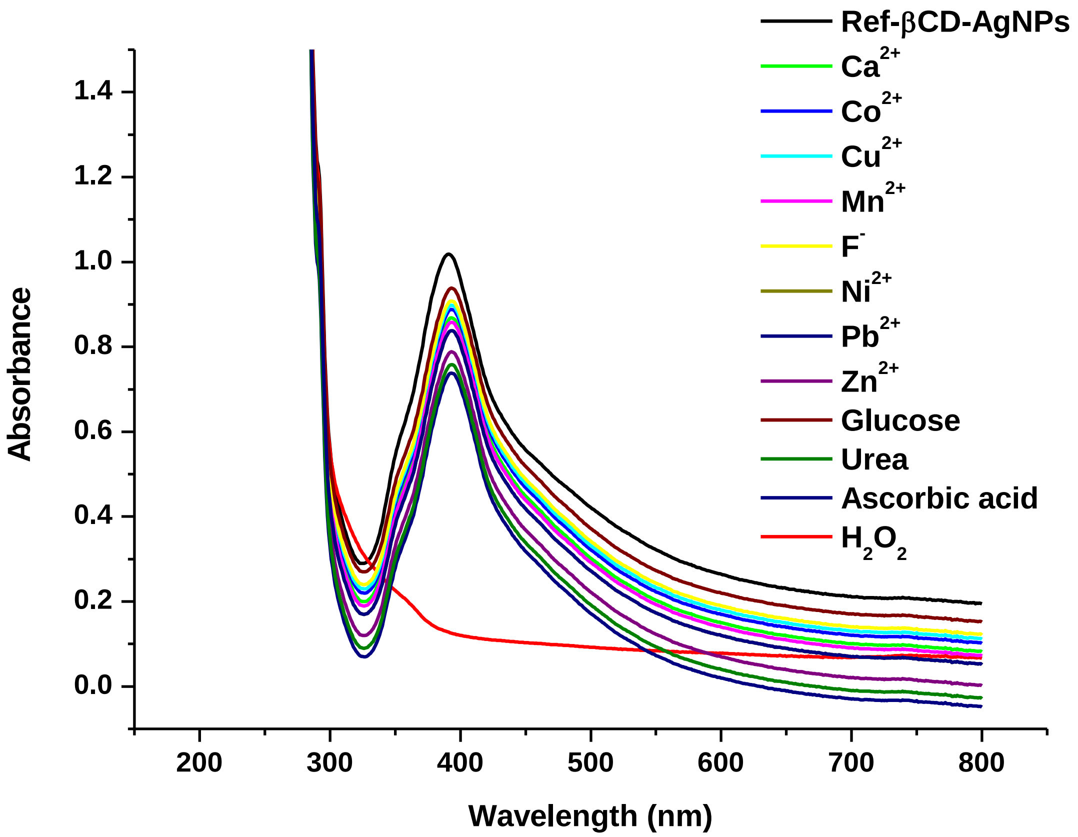

Figure S2. LSPR absorbances of Algae-AgNPs reacted with potential interferent equilibrium time = 3.0 min; pH_0 = 7.0, T = 25°C.

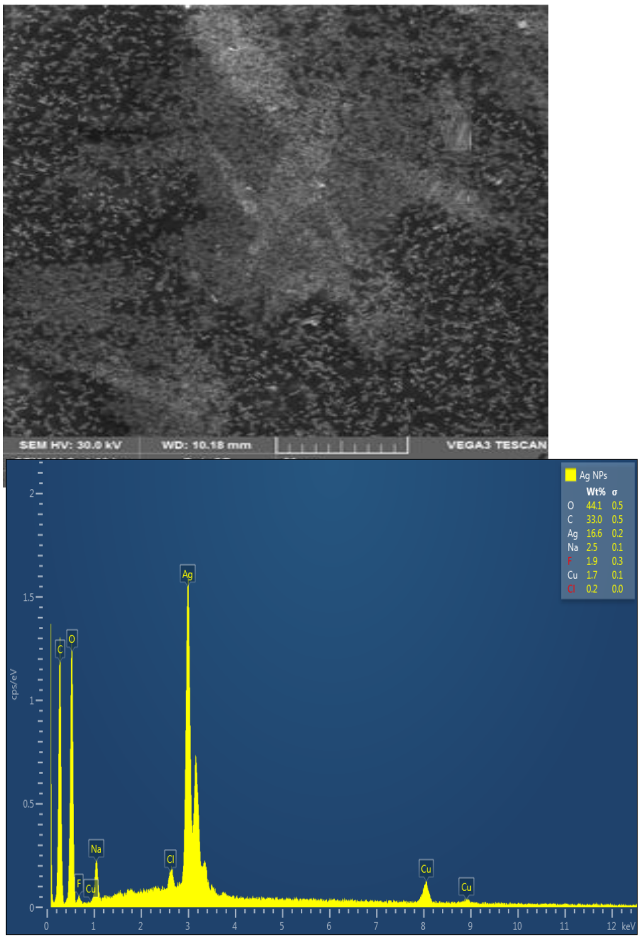

Figure S3. (**a**) Scanning Electron microscopy (SEM) and (**b**) Energy Disperse Spectroscopy (EDS) of ***βCD-AgNPs.***

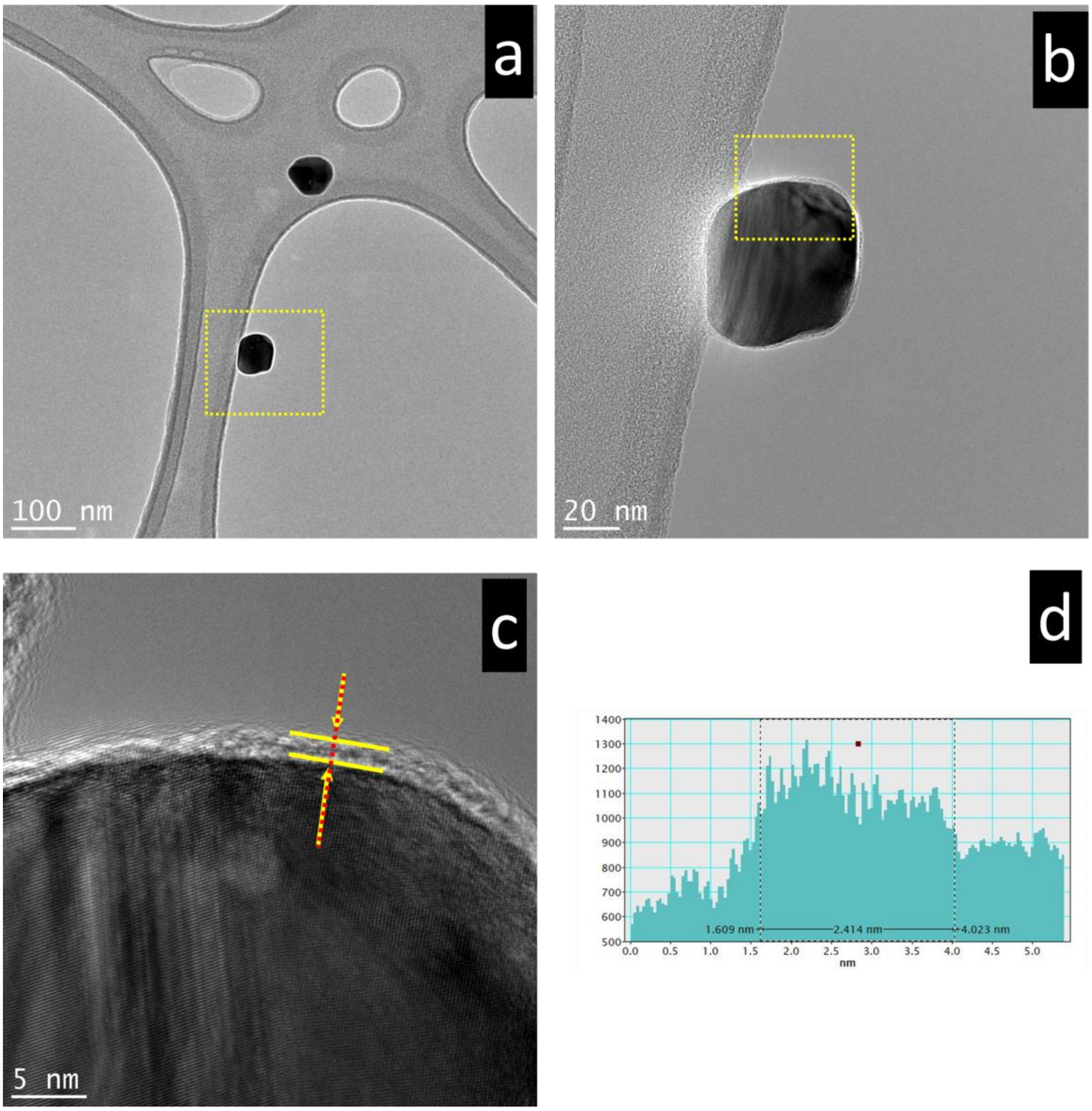

Figure S4. (**a**–**b**) TEM micrograph of a typical βCD-AgNPs. The dotted rectangle indicates the region of interest (ROI) used for the analysis. (**c**) A 2.4 nm βCD macromolecule coating is indicated by the lines and arrows in the figure. The intensity profile along the dotted line in (**c**) is shown in (**d**), indicating 2.4 nm thickness. The ROI and profile were realized using the Gatan digital micrograph platform.

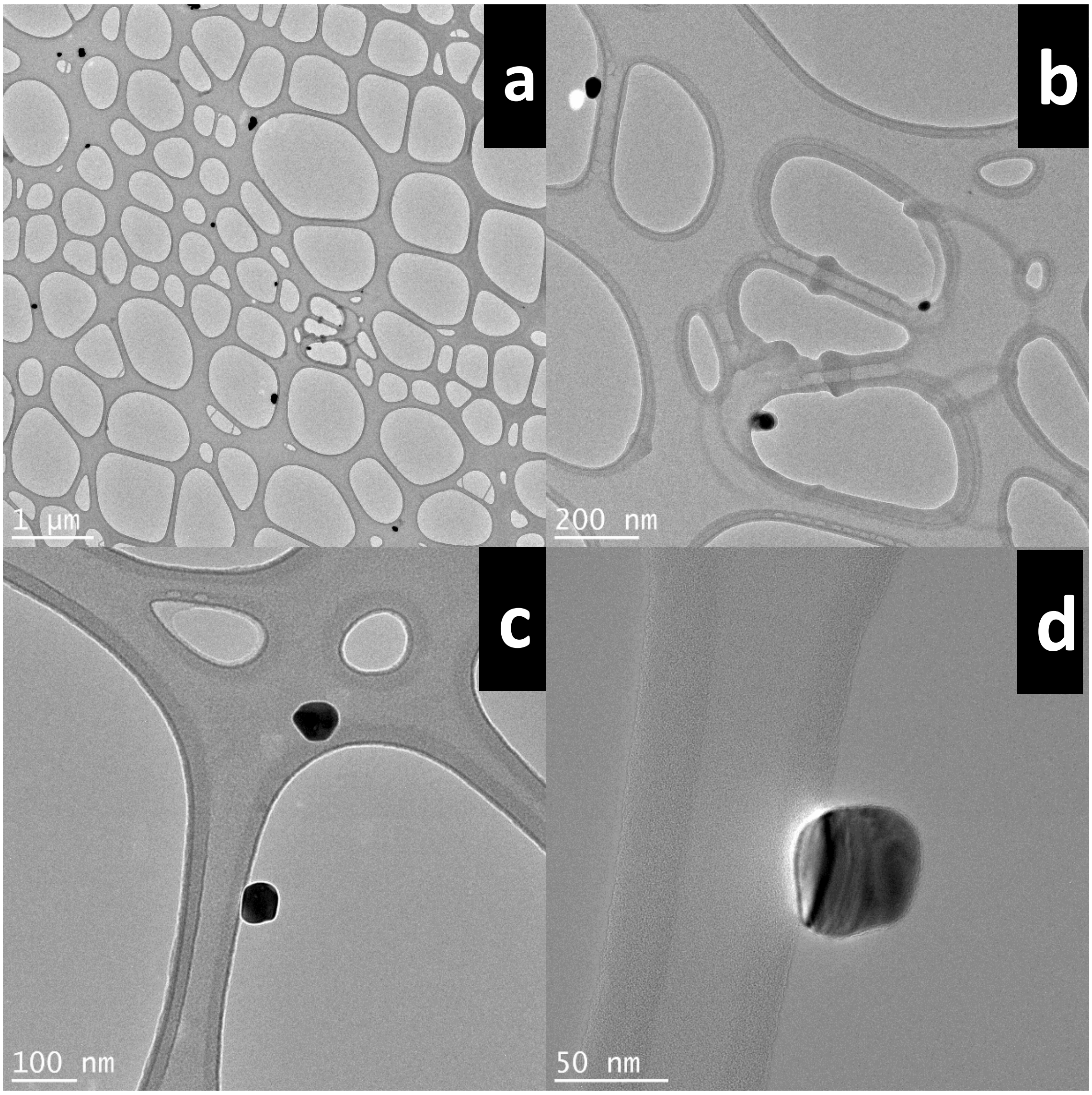

Figure S5. TEM micrograph of βCD-AgNPs, with (**a**) 1.0 µm, (**b**) 50 nm, (**c**) 100 nm, and (**d**) 200 nm size scale bars taken at different magnification.
